# Precocious *Eimeria magna* transgenically expressing RHDV P2 subdomain induces immune responses in rabbits

**DOI:** 10.1038/s41541-025-01223-9

**Published:** 2025-07-24

**Authors:** Wenxuan Chen, Jingxia Suo, Jiahua Kong, Chunxia Lu, Xiaomin Ge, Fang Yu, Xinming Tang, Xun Suo, Xianyong Liu

**Affiliations:** 1 State Key Laboratory of Veterinary Public Health and Safety; Key Laboratory of Animal Epidemiology and Zoonosis of Ministry of Agriculture, National Animal Protozoa Laboratory & College of Veterinary Medicine, China Agricultural University, Beijing, China; 2 Department of Basic Veterinary Medicine, College of Veterinary Medicine, China Agricultural University, Beijing, China; 3 Key Laboratory of Animal Biosafety Risk Prevention and Control (North) of MARA, Institute of Animal Sciences, Chinese Academy of Agricultural Sciences, Beijing, China

**Keywords:** Live attenuated vaccines, Live attenuated vaccines, Parasitic infection

## Abstract

Rabbit coccidiosis and rabbit haemorrhagic disease (RHD) pose major threats to the rabbit industry, causing significant economic losses. Developing a multivalent vaccine to concurrently protect rabbits against *Eimeria* and RHDV infections would provide dual protection through a single immunization protocol. Here, we utilized a precocious line of *E. magna* (EmagPWT) as a vaccine vector to express P2 subdomain of RHDV capsid protein VP60. We constructed three transgenic parasites expressing (i) RHDV1-P2 subdomain, (ii) RHDV2-P2 subdomain, and (iii) 2 copies of P2 subdomains from both RHDV1 and RHDV2. We found that all transgenic parasites elicited detectable neutralizing antibodies and robust mucosal immune response following secondary immunization. In conclusion, our results indicate genetically manipulated precocious *Eimeria* parasite expressing heterologous antigens, such as P2 subdomain, holds promise as a vector for developing a multivalent vaccine against RHD and *Eimeria* infections in rabbits.

## Introduction

Over the past decade, the rabbit industry has expanded considerably, driven by rising demand for rabbit meat and growing interest in pet ownership^1^. Additionally, rabbits serve as laboratory animals, contributing to research on human and animal health^2^. Unfortunately, diseases such as rabbit hemorrhagic disease (RHD) and coccidiosis pose serious threats to both domestic and wild rabbit populations, with outbreaks on rabbit farms can result in substantial economic losses^3–5^.

RHD is a kind of acute, septicemic, and highly fatal infectious disease caused by the rabbit hemorrhagic disease virus (RHDV), a member of *Lagovirus* genus within the Caliciviridae family. RHD first outbreak occurred in China in 1984^4^. In 2010, a new strain of RHDV was reported in France, named RHDV2/b/GI.2^6^ and China reported its first case of RHDV2 in 2020^7^. Compared to classical RHDV (RHDV1), RHDV2 poses greater risks due to its ability to infect younger rabbits and exhibit a broader host range, while it provides lower cross-protection in rabbits immunized with RHDV1 vaccines^8–12^. Consequently, there is an urgent need for RHD vaccines to mitigate the spread of both RHDV1 and RHDV2.

The RHDV capsid protein VP60 is a key protective protein for eliciting immune responses, making it a prime candidate component for the novel RHD vaccine^13–17^. The P2 subdomain, located on the outermost surface of VP60, is thought to contain critical determinants for cell attachment and antigenic variation^18^.Notably, the loop 1 within P2 is involved in virus-host interaction and serves as a major neutralization site that could induce effective immune responses^19^.

Rabbit coccidiosis is a protozoal disease caused by *Eimeria* spp., which parasitize the digestive system. Infected rabbits typically show clinical symptoms such as depression, diarrhea, weight loss, and, in severe cases, death. Current prevention and treatment strategies primarily rely on anticoccidial medications, but their efficacy is limited by emerging drug resistance, which gradually diminishes therapeutic effects, and potential residues in rabbit products. Interestingly, animals exposed to moderate doses of *Eimeria* oocyst can develop robust immune responses, demonstrating the feasibility of live oocyst vaccines^20–22^. Advances in gene editing technology have enabled the development of transgenic *Eimeria* strains expressing exogenous antigens, with studies showing that some can elicit specific immune responses against these antigens^23–26^. Given the high virulence of wild-type *Eimeria* strains, utilizing attenuated strains as vaccine candidates represents a safer and more effective approach. Based on this foundation, in the present study, we investigated the potential of *E. magna* precocious line (EmagPWT)—a strain with low pathogenicity and kept immunogenicity—as a vaccine vector for RHDV. We engineered EmagPWT to express the VP60-P2 subdomain of RHDV1, RHDV2, or both, and evaluated their immunogenicity against RHDV and *Eimeria*.

## Results

### Construction and Identification of transgenic parasites expressing P2 subdomain from RHDV1 or/and RHDV2 in precocious *Eimeria magna*

**Fig. 1:**
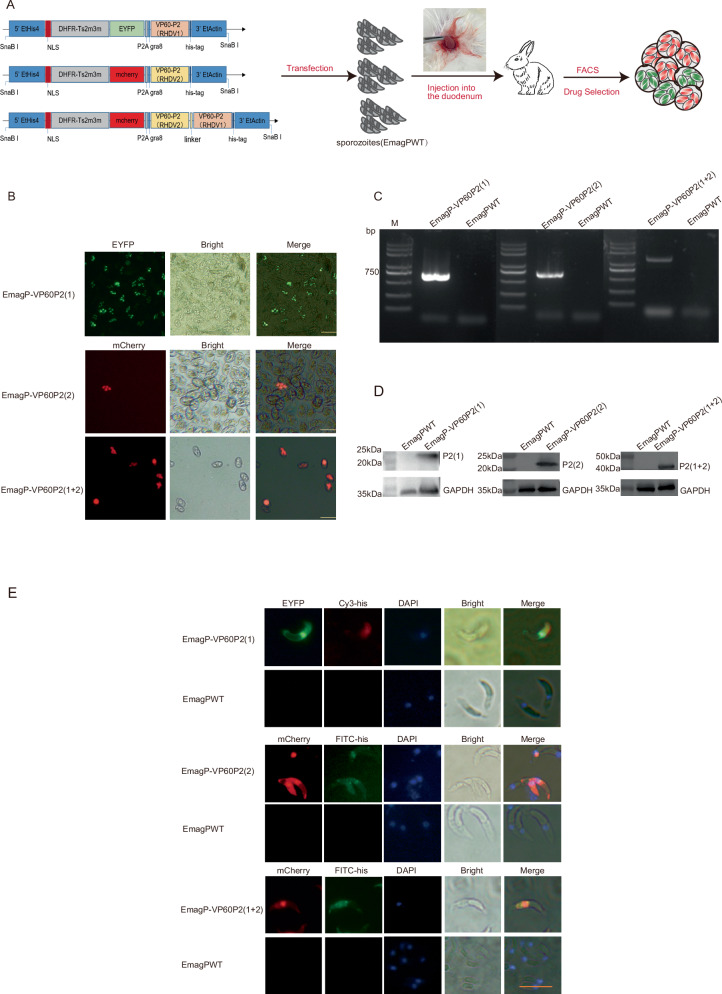
Construction and identification of transgenic parasites. **A** Diagram of transfected plasmids, and *Sna*B I was employed to linearize the plasmids. **B** The fluorescent transgenic sporulated oocysts. Scale bar = 50 μm. **C** Genomic DNA from transgenic parasites was amplified with the primers JD-F and JD-R, the target fragments were 731 bp in EmagP-VP60P2(1) and EmagP-VP60P2(2), and 1364 bp in EmagP-VP60P2(1 + 2). Genomic DNA from EmagPWT was used as a control; M, *Trans*2K Plus DNA marker. **D** P2 protein expression in the recombinant EmagPWT was confirmed via western blot analysis with rabbit anti-His-tag antibody, which detected products at approximately 22 kDa in EmagP-VP60P2(1) and EmagP-VP60P2(2), and 45 kDa in EmagP-VP60P2(1 + 2). **E** P2 expression and distribution in the transgenic parasites sporozoites was confirmed via IFA with rabbit anti-His-tag antibody. Scale bar = 10 μm.

The three plasmids, pHDE-VP60P2(RHDV1), pHDM-VP60P2(RHDV2) and pHDM-VP60P2(RHDV1 + RHDV 2) were individually transfected into EmagPWT sporozoites. Through fluorescence-activated cell sorting (FACS) and pyrimethamine selection, three stably transfected parasite populations were obtained, i.e., EmagP-VP60P2(1), EmagP-VP60P2(2) and EmagP-VP60P2(1 + 2) (Fig. 1B). The fluorescence rate for these three populations were 95%, 60% and 95%, respectively (Supplementary Table 1).

We further confirmed the successful construction of these transgenic parasites. PCR analysis verified the integration of each plasmid into the EmagPWT genome (Fig. 1C). Western blot analysis using an anti-His-tag antibody detected a specific band of approximately 22 kDa was detected in EmagP-VP60P2(1) and EmagP-VP60P2(2), whereas a specific band at approximately 45 kDa was observed in EmagP-VP60P2(1 + 2) (Fig. 1D, Supplementary Fig. 1). The IFA further evidenced that exogenous expressed P2 protein mainly localized primarily to the sporozoite cytoplasm (Fig. 1E).

### Transgenic parasites have similar biological characteristics of EmagPWT

**Fig. 2:**
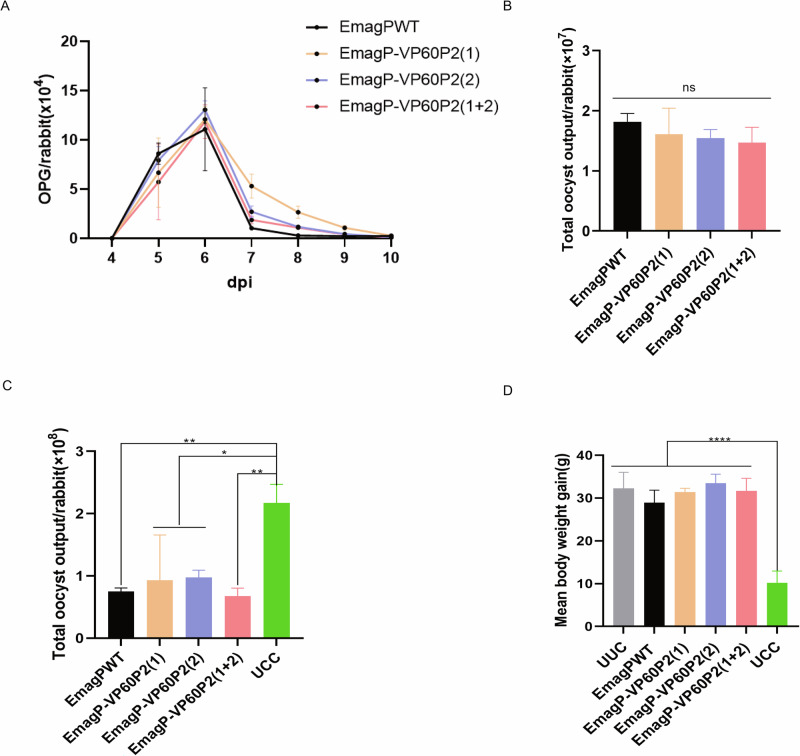
Similarity in biological characteristics between transgenic parasites and EmagPWT. **A** Daily oocysts per gram (OPG) in rabbits inoculated with 500 oocysts of either EmagPWT or transgenic parasites. **B** Total oocyst output from day 4 to 10 post-primary infection. **C** Total oocyst output from 6.5 to 14 days post-challenge with 1 × 10^4^ EmagWT oocysts. **D** Mean body weight gain measured over 14 days post-challenge. All values are presented as mean ± SEM (n = 3). Statistical significance was determined using one-way ANOVA with Tukey’s post-test; ns = non-significant, *p ≤ 0.05, **p ≤ 0.01,****p ≤ 0.0001.

The biological characteristics of *Eimeria* parasites are typically analyzed by assessing their reproductivity and immunogenicity. In this trial, we measured daily oocyst per gram (OPG) of feces in rabbits from 4 to 10 days post-infection with 500 oocysts of either EmagPWT or the transgenic parasites EmagP-VP60P2(1), EmagP-VP60P2(2) and EmagP-VP60P2(1 + 2). Fecal sample analysis revealed that all rabbits began excreting oocysts 4 days after infection, reaching a peak at 6 dpi **(**Fig. 2A**)**. Total oocyst outputs for each group indicated that three transgenic parasites presented similar reproductivity compared with that of EmagPWT (Fig. 2B). Following challenge with 1 × 10^4^ EmagWT oocysts, immunized groups showed significantly reduced oocyst output compared to UCC group (Fig. 2C). There was no significant difference in weight gain was observed among the immunized groups compared to the UUC group, but it was notably higher than that in the UCC group (Fig. 2D). In addition, rabbits in the UCC group showed signs of depression and moderate diarrhea, which were not observed in rabbits immunized with EmagPWT or transgenic parasites, demonstrating that the transgenic parasites maintain immunogenicity similar to EmagPWT. These results demonstrate that the transgenic parasites retain biological features equivalent to EmagPWT.

### Transgenic parasites elicit excellent antibody response against EmagPWT and detectable RDHV-specific humoral immune responses in rabbits

**Fig. 3:**
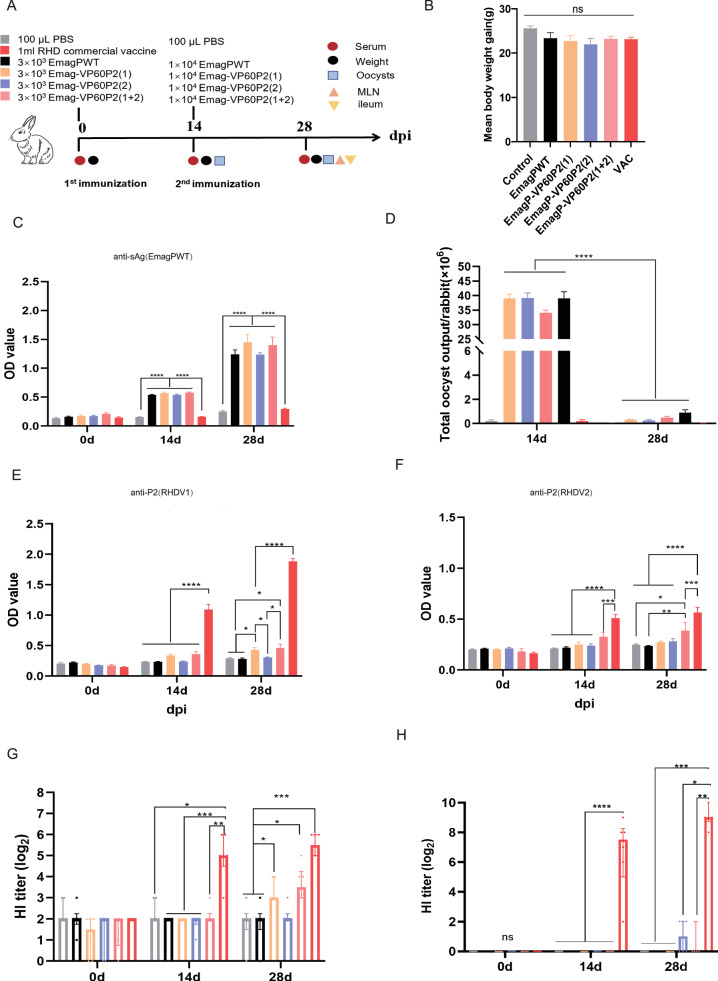
Comparison of antibody responses elicited by transgenic parasites expressing P2 from RHDV. **A** Schematic diagram of the immunization schedule and sampling timeline. **B** Body weight change of rabbits was calculated during the immunization experiment period. **C** *Eimeria*-specific IgG antibodies were detected by ELISA. **D** Total oocyst output after each immunization. **E** P2(RHDV1)-specific IgG antibodies were detected by ELISA with a recombinant P2(RHDV1) expressed in *E. coli*. **F** P2(RHDV2)-specific IgG antibodies were detected by ELISA with a recombinant P2(RHDV2) expressed in *E. coli*. **G** The HI titers of serum samples from rabbits against RHDV1(cutoff line:3). H, The HI titers of serum samples from rabbits against RHDV2(cutoff line:0). Statistical significance was determined using one-way ANOVA with Tukey’s post-test for panel **B** and two-way ANOVA with Tukey’s post-test for panels **C**–**F**, with all values presented as mean ± SEM (*n* = 6). For panels **G** and **H**, the Kruskal–Wallis test followed by the Benjamini–Hochberg post-test was used, and data are shown as median ± IQR (*n* = 6). ns = non-significant, *p ≤ 0.05, **p ≤ 0.01, ***p ≤ 0.01,****p ≤ 0.0001.

In the study of evaluating specific humoral responses induced by transgenic parasites, we employed the boosting immunization strategy in an animal trial (Fig. 3A). Measurement of rabbit weight gain revealed that the five immunized groups and the control group exhibited similar daily weight gain throughout the experimental period, indicating that the inoculation with either *Eimeria* oocysts or the RHD commercial vaccine did not affect rabbit growth (Fig. 3B). When using soluble antigens of EmagPWT (sAg) to detect sera, we found that IgG production against sAg in each *Eimeria*-immunized group was significantly higher than that in the control and VAC groups (Fig. 3C). Notably, oocyst outputs in these *Eimeria* re-immunized groups were considerably lower than those in the primary immunization group, indicating a robust immune response against the homologous *Eimeria* was produced in these rabbits **(**Fig. 3D).

To determine whether transgenic parasites elicit an RHDV-specific humoral immune response, prokaryotically expressed recombinant P2 proteins (Supplementary Fig. 2) were used for ELISA assay. When testing with rRHDV1-P2, IgG levels in the VAC group were significantly higher than those in all other groups. Following booster immunization, EmagP-VP60P2(1) and EmagP-VP60P2(1 + 2) groups showed significantly higher IgG antibody levels than the control, EmagPWT, and EmagP-VP60P2(2) groups (Fig. 3E). When coating with rRHDV2-P2, IgG levels in EmagP-VP60P2(2) and EmagP-VP60P2(1 + 2) groups showed a slight increasing trend across three time points, but EmagP-VP60P2(2) did not differ significantly from the EmagPWT and control groups. At 28 dpi, the EmagP-VP60P2(1 + 2) group exhibited higher IgG levels than the EmagPWT and control groups, yet remained significantly lower than the VAC group (Fig. 3F).

The HI assay was conducted to evaluate the capacity of serum samples to neutralize RHDV. Sera from rabbits immunized with RHD commercial vaccine displayed significantly enhanced neutralization ability against both RHDV1 and RHDV2 compared to other groups (Fig. 3G, H). Regarding RHDV1, HI titers in the sera of certain rabbits belonging to EmagP-VP60P2(1) and EmagP-VP60P2(1 + 2) groups were exceeded the critical value after the secondary vaccination, showing a significant increase relative to the control and EmagPWT groups (Fig. 3G). For RHDV2, HI titers in some sera from rabbits immunized with EmagP-VP60P2(2) and EmagP-VP60P2(1 + 2) were slightly elevated compared with those from the control, EmagPWT and EmagP-VP60P2(1) groups (Fig. 3H). These results indicated that although the levels of antibodies against exogenous antigens in rabbits immunized with the transgenic parasites were substantially lower than those elicited by the RHD commercial vaccine, but transgenic parasites still stimulated detectable RHDV-specific antibodies in rabbits.

### *Eimeria* parasites can stimulate higher cellular immune response

**Fig. 4:**
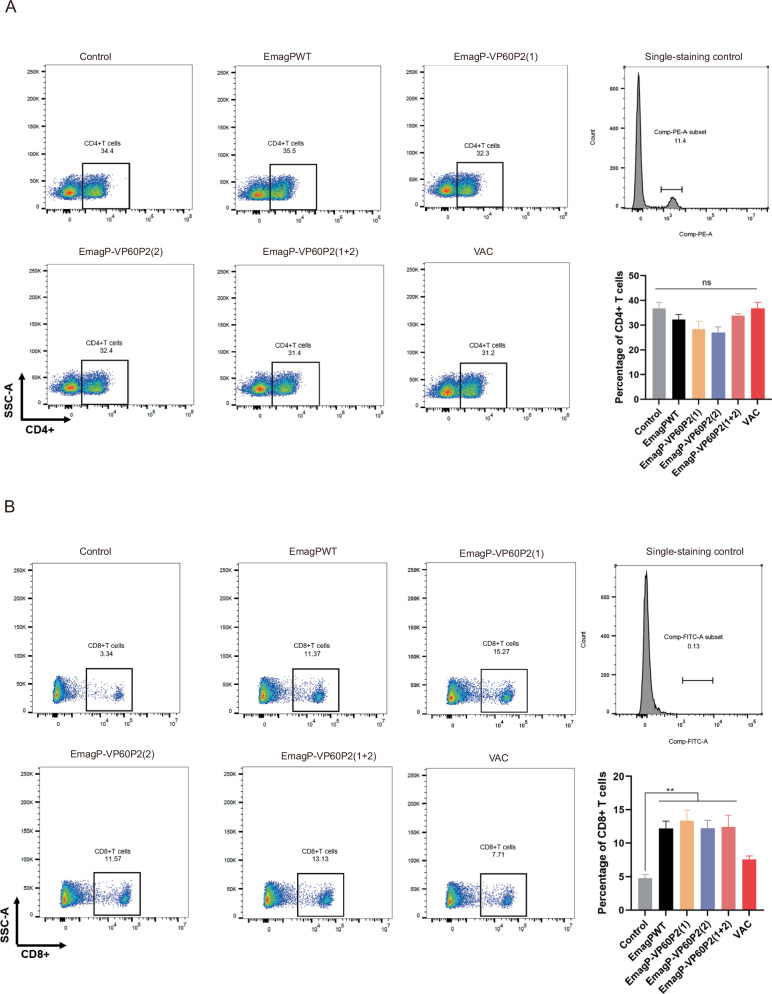
Percentage of CD4 + and CD8+ T cell subpopulations in MLN. **A** Flow cytometric analysis for expression of CD4+ T cells in MLN. **B** Flow cytometric analysis for expression of CD8+ T cells in MLN. All values are presented as mean ± SEM (*n* = 6). Statistical significance was determined using one-way ANOVA with Tukey’s post-test; ns = non-significant, **p ≤ 0.01.

*Eimeria* spp. infection the hosts can stimulate a humoral response, yet cell-mediated immunity plays a major role, in which both CD4+ and CD8+ lymphocytes are involved. In our story, the percentage of CD4+ lymphocytes in the MLN of rabbits remained consistent across all groups at 14 days after boost immunization, ranged from 27% to 40% (Fig. 4A). Notably, we observed the percentage of CD8+ lymphocytes from rabbits immunized with *Eimeria* parasites was significant higher compared to the control group (4.76%), but statistically this value did not differ from that in the VAC group (7.51%) (Fig. 4B). Since the ileum is a major parasitic site of these parasites, we performed immunofluorescence analysis on ileum sections. The results suggested that CD4+ and CD8 + T cells infiltrated this region, with a slightly more prominent tendency in the *Eimeria*-parasitized immunized group (Supplementary Fig. 3). Our current observations showed that a marked elevation of CD8+ lymphocytes in the MLN and evidence of CD4+ and CD8 + T cell involvement in the ileum following re-immunization with *Eimeria* parasites, suggesting they can stimulate local mucosal immune responses.

## Discussion

The development of novel vaccines is an urgent need for controlling infectious diseases in livestock and poultry, especially in the context of reducing and replacing the use of antibiotics globally. For the prevention of rabbit hemorrhagic disease and coccidiosis, the most common diseases in rabbits, single-dose vaccination is the ideal approach. To test this hypothesis, we attempted to develop recombinant vaccines using *Eimeria* parasites as vectors for expressing heterologous antigen^23,24,27^. In this study, we constructed three transgenic precocious line of *E.magna* expressing the P2 subdomain of rabbit hemorrhagic disease virus (RHDV1, RHDV2). We further demonstrated that immunization with these transgenic parasites elicited detectable RHDV-specific antibody responses in rabbits. These results suggest that these transgenic parasites hold promise as RHD vaccine candidates for rabbits, a finding that merits further investigation.

RHD, caused by RHDV1 and RHDV2, is an extremely contagious and fatal disease affecting rabbits and hares. Effective strategies to prevent and control RHDV transmission are urgently needed. Nevertheless, the challenge is complicated by the absence of a functional in vitro system for RHDV propagation, substantially impeding virological studies and vaccine development^14^. For a long time, commercial vaccines against RHD have been prepared by chemically inactivating liver tissue suspensions from artificially infected rabbits. However, this type of vaccine raises concerns regarding animal welfare and the risk of viral dissemination during the vaccine preparation process. Genetically engineered vaccines, characterized by their high efficacy and safety, have become an important research direction for RHD vaccines. The capsid protein VP60, serving as the critical immunoprotective antigen, plays an indispensable role in stimulating the immune response against viral infections. Multiple heterologous expression platforms and recombinant viral vectors have been engineered to generate VP60 protein derivatives^13,16,28,29^. The majority of these vaccines are administered by injection, which is labor-intensive and may cause stress in animals^30,31^. Consequently, opting for an orally administered vaccine may represent a more advantageous alternative. The P2 subdomain, located at the outermost surface exposed region of the RHDV capsomere, has been confirmed that contain a flexible loop (aa 304–314) that is involved in host-virus interactions and serves as a primary neutralization site that can induce effective host immune responses^19^. Additionally, two epitopes (aa 326–331 and 338–342) within the P2 subdomain may be crucial to the antigenic structure of VP60 and the interaction of RHDV and HBGA^32^. These results illustrate the importance of the P2 subdomain. Therefore, P2 was selected as an immunogen for vaccine development in this study.

*Eimeria* spp. are promising candidates for oral vaccine vectors due to their numerous advantages. These parasites have a large genome, which facilitates the efficient incorporation of heterologous genes. Additionally, they can post-translationally modify heterologous proteins to preserve their native structure and immunogenicity. *Eimeria* exhibits strong host specificity, and the subcellular structure known as the parasitophorous vacuole (PV) considerably hinders the integration of exogenous genes into the host genome, thereby ensuring the biosafety of this approach. The development of genetic manipulation techniques for *Toxoplasma gondii* and *Plasmodium* has provided valuable methodologies for *Eimeria*^33–36^. Currently, stable transfection platforms for certain *Eimeria* species are maturing, with successful transfections achieved in wild-type strains such as *E. magna, E. tenella, E. intestinalis, E. mitis*, etc. Heterologous antigens expressed by these recombinant *Eimeria* can be recognized by the host immune system, eliciting specific immune responses^24,25,37,38^. Compared with the wild-type strains, the precocious strains have a shorter prepatent period and reduced pathogenicity while retaining comparable immunogenicity^21,39^. This reduces the time required to generate stable transgenic *Eimeria* and avoids the risks associated with the strong pathogenicity of wild-type strains.

In this study, we successfully obtained three transgenic precocious *E. magna* expressing P2 subdomain from RHDV1 and RHDV2 for the first time. The transgenic P2 displayed successful integration and expression within both transgenic parasites, with the P2 protein predominantly localized in the cytoplasm of sporozoites (Fig. 1). We observed that each transgenic parasite exhibited good immunogenicity, providing excellent protection against *E. magna* infection (Fig. 2), indicated that the insertion of P2 gene did not change the basic biological characteristics of EmagPWT. Therefore, these three transgenic parasites serve as valuable materials for further studies.

A robust immune response is crucial for *Eimeria* as a vaccine vector. Although we did not observe a significant antibody response after primary immunization, we found that 14 days post-secondary immunization, the EmagP-VP60P2(1) and EmagP-VP60P2(1 + 2) groups exhibited positive HI titers against RHDV1. Meanwhile, some rabbits in the EmagP-VP60P2(2) and EmagP-VP60P2(1 + 2) groups showed detectable HI titers against RHDV2. However, the antibody levels in rabbits receiving transgenic parasites were still lower than those in the RHD commercial vaccine group (Fig. 3). Nonetheless, these transgenic parasites triggered a specific humoral immune response to heterologous antigens in rabbits, which differed from those observed in the control and EmagPWT groups. Furthermore, a notable local immune response was observed in the MLN and ileum. Limited data exist regarding the immune response of rabbits to coccidia. Most previous studies focused on primary infection, leaving a gap in the understanding of secondary infections. Renaux et al. (2003) reported an increased proportion of CD8+ lymphocytes in the MLN and intestines of rabbits at 14 and 21 days post-infection with *E. intestinalis*^40^. Conversely, Pakandl et al. (2008) reported no significant change in the proportion of CD8+ lymphocytes in the MLN^41^. Subsequently, they refined the intestinal cell isolation method and found that both CD4+ and CD8+ lymphocytes predominantly infiltrated the intestinal lamina propria. Our results are generally consistent with theirs, showing that CD4+ lymphocytes in the MLN did not change significantly after immunization with *Eimeria*, whereas the proportion of CD8+ lymphocytes was elevated compared to both the control group and the RHD commercial vaccine group (Fig. 4). Immunofluorescence analysis of ileum tissue sections suggested a slightly increase in the presence of CD4+ and CD8 + T cells within the ileum of rabbits belonging to the *Eimeria*-immunized group (Supplementary Fig. 3). Although we were unable to assess the RHDV-specific cellular immune response due to constraints, our findings confirmed that the transgenic EmagPWT can elicit an immune response against exogenous antigens and has the potential as a vaccine carrier.

Previous research has indicated that loop 1 within the P2 subdomain forms an epitope of RHDV, and peptide segments derived from this loop are sufficient to elicit a protective response against 256 HA titer of RHDV challenge^19^. Although we detected RHDV-specific antibodies in some rabbits immunized with transgenic parasites, these antibodies failed to provide effective protection in challenge experiments (data not shown). Results showed that EmagP-VP60P2(1) and EmagP-VP60P2 (1 + 2) transgenic parasites provided partial protection for some rabbits. We hypothesize that this may be attributed to insufficient expression levels of the P2 protein in transgenic parasites. By contrast, the baculovirus expression vector system—one of the major protein expression platforms—offers advantages such as high-yield protein production and strong immunogenicity. In contrast, *Eimeria* faces a challenge: limited antigen production, which may be insufficient to elicit an adequate immune response.

For future studies, transgenic *Eimeria* vaccines need to be further optimized to enhance their efficacy in eliciting robust immune responses against heterologous antigens. In addition to increasing heterologous protein expression, exploring delivery methods or different adjuvants could also amplify immune responses. Investigations involving intracellular parasites, specifically *Toxoplasma gondii*^42^, *Leishmania major*^43^, *Trypanosoma cruzi* ^44^, *Eimeria acervuline*^26^ and *Eimeria tenella*^27^ have provided key insights. These studies examined the ability of heterologous antigens targeted toward distinct subcellular compartments to stimulate immune responses in the host. Findings revealed that antigens secreted into the extracellular environment, referred to as secretory antigens, were effectively presented to CD8 + T cells, whereas neither cytosolic nor transmembrane proteins primed these immune cells effectively. This dichotomy highlights selective antigen presentation pathways critical for eliciting robust cellular immunity. Meanwhile, multiple studies have demonstrated that molecular adjuvants expressed by *Eimeria* significantly enhance the immunogenicity of recombinant parasites. For instance, transgenic *E. tenella* expressing *E. maxima* profilin boosts the parasite’s antigen-specific immune responses and improves resistance to *E. tenella* infection^45^. Additionally, transgenic *E. necatrix* expressing chicken interleukin-1β (chIL-1β) enhances immunogenicity and mucosal immunity^46^. These findings indicate that genetic modification of *Eimeria* to elevate the immune responses is a viable strategy. Collectively, these insights outline a promising vaccine design strategy utilizing transgenic *Eimeria* systems in rabbits. This approach could simultaneously enable effective prevention against both RHD and rabbit coccidiosis, thereby improving rabbit population health, though significant challenges remain.

## Methods

### Parasites and Animals

The parental wild-type strain and precocious line of *Eimeria magna* (EmagWT and EmagPWT) are preserved in our laboratory at China Agricultural University. The procedures for oocysts passage, collection, purification, and sporulation have been previously described^47^.

All rabbits used in this study were sourced from a rabbit farm at Zhuozhou Experimental Station of China Agricultural University. They were housed in isolators and provided with coccidia-free pellet feed and water *ad libitum*. All experimental protocols received approval from the Animal Ethics Committee of China Agricultural University (Approval No.AW11214202-1-01). We ensured the well-being of rabbits throughout the study. The rabbits are housed in a stress-free environment, free from excessive light and noise. We performed daily observations of the rabbits during all experimental procedures.

### Anesthesia and Euthanasia

Anesthesia was induced using a combination with Zoletil 50 and Sumianxin II. Before anesthesia, rabbits were fasted for 12 hours and water-deprived for 4 hours; then they were weighed to calculate the injection dosage. First, 0.01 ml/kg of Zoletil 50 was injected intramuscularly, followed immediately by 0.01 ml/kg of Sumianxin II. Subsequently, the depth of anesthesia was evaluated according to postural reflex, muscular tone, palpebral reflex, corneal reflex, laryngeal reflex, ear pinch, and digital (pedal) reflexes. If the rabbit had no righting reflex, showed muscle relaxation, stable breathing, and no obvious response to external stimuli, indicating that it has entered the state of anesthesia. For euthanasia, deeply anesthetized rabbits underwent cardiac exsanguination. This method ensured rapid and humane termination while maintaining profound anesthesia throughout the procedure. All protocols were designed to minimize animal stress and discomfort in accordance with ethical guidelines for laboratory animal use.

### Plasmid construction

Based on the VP60 sequence of RHDV1 (GenBank Accession number: AY269825.1) and RHDV2 (GenBank Accession number: MT383749), the 287 to 483 amino acids were selected (P2 subdomain, aa287-449 and 467-483). The sequence was codon-optimized (http://genomes.urv.es/OPTIMIZER/) according to codon usage bias in *E. tenella* (https://www.kazusa.or.jp/codon/). An *Age*I restriction site (ACCGGT) was added at the start of the optimized sequence, while a His-tag and the termination codon (GATTACAAGGATGACGACGATAAGTGA) as well as *SacII* (CCGCGG) were appended at the end. The synthesis was performed by Beijing Ruibiotech Co., Ltd (Beijing, China). (i) for plasmid pHDE-VP60P2(RHDV1), mainly includes histone 4 promoter from *E. tenella*, 3′ untranslated region of actin from *E. tenella*, and DHFR-Ts2m3m (a pyrimethamine resistance gene from *T. gondii*). It also included a porcine teschovirus-1 2 A peptide (P2A) to cleave two contiguous proteins between the enhanced yellow fluorescent gene (EYFP) and P2(RHDV1) gene, along with a *T. gondii* gra8-derived signal sequence (gra8ss) to enhance P2 secretion, followed by a His-tag. (ii) for plasmid pHDM-VP60P2(RHDV2), on the basis of pHDE-VP60P2(RHDV1), it replaced EYFP with red fluorescent reporter gene (mCherry) and P2(RHDV1) with P2(RHDV2). (iii) for plasmid pHDM-VP60P2(RHDV1 + RHDV2), based on plasmid pHDM-VP60P2(RHDV2), flexible linker sequence and P2(RHDV1) sequence were added after P2(RHDV2) (Fig. 1A). All fragments were amplified using the Q5 High-Fidelity DNA Polymerase (New England BioLabs, Inc.) to minimize mutation frequency and were subsequently ligated via Seamless Cloning and Assembly Kit (TransGen Biotech, Beijing, China). A list of primers employed in this study is provided in Supplementary Table 2.

### Transfection and selection of transgenic parasites

For stable transfection of EmagPWT, 10 million sporozoites, which had been freshly extracted and purified using DE-52 cellulose column^48^, 50–60 μg linearized DNA plasmid, and 5 μL *Sna*BI restriction enzyme were electroporated using a nucleofector (Program U-033, AMAXA, Switzerland)^49^. The transfected sporozoites were resuspended in DMEM and surgically injected into the duodenum of a 35-day-old rabbit. Oocysts were subsequently harvested from feces collected 5-8 days post-infection. Transgenic oocysts were selected by fluorescence-activated cell sorting (FACS; MoFlo Cell Sorter, Dako-Cytomation, Fort Collins, CO, USA) and pyrimethamine^50^, followed by propagation of the next generation.

### DNA extraction and identification of transgenic parasites

After several generations of transgenic parasites selection, genomic DNA from oocysts of EmagPWT and transgenic parasites using DNA extraction kit (Tiangen Biotech, Beijing, China) to verify whether the P2 gene was inserted into the coccidia genome. Exogenous gene amplification was then performed using these DNAs as templates with specific primers JD-F(5’-GGTGTTCGTGGTCTTCG-3’) and JD-R(5’-GTTTCGCAAGTCATGAAC-3’). Genomic DNA from EmagPWT was used as a negative control.

### Western blot

Soluble proteins were extracted from the oocysts to confirm successful expression of the P2 gene in these transgenic parasites^48^. Protein from EmagPWT served as a negative control. The protein supernatant was then subjected to SDS-polyacrylamide gels (SDS-PAGE) and transferred onto PVDF membranes for further analysis. After blocking with 5% skimmed milk in PBST (0.05% PBS-Tween) for 1 h at room temperature (RT), the membranes were incubated for 1 h at RT with primary antibodies diluted in primary antibody diluent buffer (Beyotime): rabbit anti-His-tag antibody (1:2000, Gene-Protein Link Inc.) or mouse anti-GAPDH antibody (1:2000, Proteintech). Following three washes with PBST, the membranes were incubated with HRP-conjugated goat anti-rabbit IgG (H + L) or HRP-conjugated goat anti-mouse IgG (H + L) (1:2000, Proteintech) diluted in the same diluent buffer (Beyotime) for 1 h at RT, followed by three additional PBST washes. Protein bands were visualized using enhanced chemiluminescence (ECL) and automatic chemiluminescence imaging analysis system (Tanon) for chemiluminescence detection.

### Indirect Immunofluorescence Assay (IFA)

To determine the subcellular localization of P2 in the sporozoites of transgenic parasites, IFA was performed as previously described^27^. Briefly, 1 × 10^6^ purified sporozoites of transgenic parasites or EmagPWT were adhered to poly-Lysine-coated slides for 15 min at RT. Samples were permeabilized with 0.2% Triton X-100 for 5 min at RT, followed by three 5-min washes in PBS (pH 7.2, 300 rpm); samples were blocked with 1% BSA for 30 min at RT. Slides were then incubated with rabbit anti-His-tag antibody (1:200, Gene-Protein Link Inc.) diluted in PBS for 1 h at RT. Unbound antibody was removed by three 5-min washes in PBS (300 rpm). Cy3 or FITC-conjugated goat-anti rabbit IgG (1:2000, Proteintech) diluted in PBS was applied for 1 h at RT in the dark, followed by three 5-min PBS washes (300 rpm). Nuclei were counterstained with DAPI (1 μg/mL) for 3 min at RT, with three 5-min PBS washes. All steps from secondary antibody incubation were performed in the dark. Imaging was performed using an Olympus fluorescence microscope (Olympus Corporation, Japan).

### Reproductivity and Immunogenicity of transgenic parasites

To evaluate the biological characteristics of transgenic parasites, we investigated their reproductivity and immunogenicity compared with EmagPWT. Eighteen 35-day-old rabbits (regardless of sex) were randomly assigned to six groups. Rabbits in unimmunized and unchallenged control group (UUC) and unimmunized but challenged control group (UCC) were inoculated orally with 100 μL PBS. Rabbits in the EmagPWT, EmagP-VP60P2(1), EmagP-VP60P2(2) and EmagP-VP60P2(1 + 2) groups were orally inoculated with 500 sporulated oocysts per rabbit. Fecal oocyst outputs were quantified daily from days 4 to 10 post-infection (dpi) using the McMaster technique^47^, and the total number of oocysts excreted during this period was recorded. At 14 dpi, all groups except the UUC group were orally challenged with 1 × 10⁴ EmagWT sporulated oocysts. For the challenged groups, fecal samples were collected and quantified from 6.5 to 14 days post-challenge to determine the total oocyst excretion during this period, while body weight gain was calculated for each group from challenge to 14 days post-challenge.

### Immunological experiment in rabbits

35-day-old rabbits (regardless of sex) were randomly divided into six groups (*n* = 6 per group): (1) control (unvaccinated), (2) VAC (RHD commercial baculovirus vector bivalent vaccine for RHDV1 and RHDV2, Qilu Animal Health Products Co., Ltd., 1 mL subcutaneously), (3) EmagPWT, (4) EmagP-VP60P2(1), (5) EmagP-VP60P2(2) and (6) EmagP-VP60P2(1 + 2). Rabbits in groups 3–6 received 3 × 10^3^ fresh oocysts orally for primary immunization, followed by a booster immunization with 1 × 10⁴ sporulated oocysts on day 14 post-prime. Serum samples were collected on day 0 (pre-immunization) and at 14-day intervals post-immunization for antibody detection. Fecal samples were collected 14 days post-immunization for oocyst counting. All rabbits were humanely euthanized on day 28 for collection of mesenteric lymph nodes and ileum sections for flow cytometry and immunofluorescence analysis, respectively. The immunization schedule and samples collection are illustrated on a time axis (Fig. 3A).

### Preparation of mesenteric lymph node (MLN) cell suspensions

MLNs were excised and immediately placed in ice-cold PBS. After careful removal of surrounding adipose and connective tissues using sterile surgical scissors, the MLN were mechanically dissociated by pressing through a 70 μm cell strainer with the plunger of a 5 mL syringe. The cell suspension was collected in RPMI-1640 medium containing 2% (v/v) FBS and centrifuged at 400 × *g* for 5 min at 4 °C, followed by three washes in the same medium. Cells were resuspended in PBS containing 2% FBS and prepared for flow cytometric analysis.

### Flow cytometry

Prior to immunostaining, cell viability was assessed using the LIVE/DEAD™ Zombie UV Fixable Viability Kit (BioLegend) according to the manufacturer’s protocol. Cells were then washed three times with staining buffer (Beyotime) by centrifugation at 400 × *g* for 5 min at 4 °C followed. Fc receptor blocking was performed with 10% normal goat serum (Absin) for 30 min at 4 °C. Cell surface staining was performed with mouse anti-CD8 (1:100, Novus) or rabbit anti-CD4 (1:100, Novus) diluted in staining buffer for 30 min at 4 °C. Cells were washed three times with staining buffer to remove unbound primary antibodies. The second step involved incubation with FITC-conjugated goat anti-mouse IgG (1:200, Proteintech) or FITC-conjugated goat anti-rabbit IgG (1:400, Proteintech) diluted in the same staining buffer for 20 min at 4 °C in the dark. After incubation, cells were washed three times with staining buffer. Appropriate negative controls (secondary antibody-only samples without primary antibody) and unstained samples were included in all experiments. The percentages of CD4+ and CD8+ T cells were acquired using the BD Fortessa flow cytometer and analyzed with FlowJo software (v10.8.1.). Cells were first gated on FSC-A/SSC-A to exclude debris, then singlets were selected through FSC-H/FSC-A gating, followed by live cell isolation using a fixable viability dye. Subsequently, CD4 + T or CD8 + T cells were gated from the lymphocyte population. Non-specific binding signals from secondary antibodies were subtracted from the anti-CD4 or anti-CD8 antibody signals to normalize data.

### Enzyme-linked immunosorbent assay (ELISA)

Serum IgG levels against EmagPWT and P2 proteins were quantified by indirect ELISA. Briefly, 96-well microplates were coated overnight at 4 °C with 100 μL/well of either 2 µg/mL EmagPWT soluble protein or 5 µg/mL recombinant P2 proteins of RHDV1 (rRHDV1-P2) and RHDV2 (rRHDV2-P2), expressed as previously described^51^, and purified with BeyoGold™ His-tag Purification Resin (Beyotime) according to the manufacturer’s instruction. After three washes with 300 μL/well PBST, plates were blocked with 5% skimmed milk in PBST at 37 °C for 1 h. Following three washes, serum samples (100 μL/well), diluted 1:100 in 2% skimmed milk in PBST, were incubated for 1 h at 37 °C. After three washes, the plates were incubated with 100 μL/well of HRP-conjugated goat anti-rabbit IgG antibody (1:2000; Proteintech) for 1 h at 37 °C. Following final washes, the reaction was developed with 100 μL/well TMB substrate (Macgene) and plates were incubated in the dark for 10 min at RT for exactly 10 min at RT in the dark and stopped with 0.5 M sulfuric acid (50 μL/well). Optical density (OD) was measured at a wavelength of 450 nm using a microplate reader (Biotek, USA). Each serum sample was measured three times, and mean OD values were calculated.

### Hemagglutination inhibition (HI) assay

We performed an HI assay to determine the neutralization antibody titers against RHDV1 and RHDV2 in serum samples. The sera for RHDV1 testing were inactivated at 56 °C for 30 min, while the sera for RHDV2 testing were then treated with a kaolin solution. Serial two-fold diluted serum samples (25 μL) and standardized antigen containing 4 hemagglutination units (25 μL) were added to the 96-well V-bottom titration plate except for the last row which functioned as a control. After incubation at 37 °C for 35 minutes, 25 μL of 1% human O-erythrocyte suspension was added to each well, followed by a 2-hour incubation at 4 °C. Additional positive sera, negative sera, erythrocytes, and antigen were used as controls. The maximum serum dilution resulting in complete inhibition was considered the endpoint.

### Section and Immunofluorescence assay of ileum

Fresh ileum tissue was fixed with 4% paraformaldehyde, followed by paraffin embedding and sectioning. The slides were deparaffinized and rehydrated. Antigen retrieval was achieved by boiling the samples in Tris EDTA (pH 9.0) using a pressure cooker. A fluorescence staining kit (Hunan Aifang Biological Technology, China) based on the tyramide signal amplification technology was used according to the manufacturer’s instructions. Primary antibodies, including mouse anti-CD8 and rabbit anti-CD4, were purchased from Novus and used at a 1:50 dilution.

### Statistical analysis

Data were analyzed using suitable statistical methods where indicated. Data visualization and statistical computations were performed with GraphPad Prism 9.5.1 software. A P value of less than 0.05 was considered statistically significant.

## Supplementary information

Supplementary Information

## Data Availability

The original contributions presented in the study are included in the article/Supplementary Materials. Further inquiries can be directed to the corresponding author.
