# Supplementary material for: Precocious Eimeria magna transgenically expressing RHDV P2 subdomain induces immune responses in rabbits
Source: NPJ Vaccines. 2025 Jul 24;10:167. doi: 10.1038/s41541-025-01223-9 (PMC12290045; doi:10.1038/s41541-025-01223-9)
Supplement: Supplementary file 1 — Supplementary Information [file 41541_2025_1223_MOESM1_ESM.pdf]

Supplementary Table 1. Propagation of transgenic EmagP-VP60P2(1), EmagP-VP60P2(2), EmagP-VP60P2(1+2).

	Passage	Fluorecent Oocysts(%)	Selection Strategy
	1st	14.8	-
	2nd	15.6	Drug
	3rd	50.8	Drug+FACS
	4th	65.2	Drug+FACS
EmagP-VP60P2(1)	5th	42.0	Drug+FACS
	6th	40.1	Drug+FACS
	7th	84.5	Drug+FACS
	8th	87.0	Drug+FACS
	9th	＞95	Drug+FACS
	10-15th	＞95	-

	Passage	Fluorecent Oocysts(%)	Selection Strategy
	1st	0.1	-
	2nd	4.2	Drug+FACS
	3rd	21.0	Drug+FACS
	4th	21.8	Drug+FACS
EmagP-VP60P2(2)	5th	41.7	Drug+FACS
	6th	65.1	Drug+FACS
	7th	47.2	Drug+FACS
	8th	60.0	Drug+FACS
	9th	60.0	Drug+FACS
	10-15th	60.0	-

	Passage	Fluorecent Oocysts(%)	Selection Strategy
	1st	32.7	-
	2nd	59.6	Drug+FACS
	3rd	68.2	Drug+FACS
	4th	85.4	Drug+FACS
EmagP-VP60P2(1+2)	5th	60.0	Drug+FACS
	6th	82.0	Drug+FACS
	7th	88.6	Drug+FACS
	8th	> 95	Drug+FACS
	9th	> 95	Drug+FACS
	$10\text{-}15^{th}$	> 95	-

Supplementary Table 2. Details of the specific primer sequences used for PCR experiments.

Primers	Sequence(5'-3')
P2（RHDV1）-F	CTTTGACCGGTATGTGGAGCAG
P2（RHDV2）-R	CCAATCGAATTCCCGCGGTCAGTGGTG
Pro-EYFP-F	AGACTACAGTGAACGCGTA
Pro-EYFP-R	GCTGCTCCACATACCGGTCAAAGGACCGTTCA
actin-1F	CACCACCACCACCACCACTGACCGCGGGAATTCGA
actin-R	TACGCGTTCACTGTAGTCTTGCTT
P2A-P2（RHDV2）-F	GACGAGCTGTACAAGGGTACCGGAAGCGGA
P2A-P2（RHDV2）-R	ATGGTGATGGTGATGATGTGTGCCGTTTGTGCTG
actin-2F	CATCATCACCATCACCATTGACCGCGGGAATTCGA
mcherry-F	GCCACCATGGTGAGCAAGGGCGAG
mcherry-R	TACCCTTGTACAGCTCGTCCAT
Pro-DHFR-F	AGACTACAGTGAACGCGTA
Pro-DHFR-R	CTTGCTCACCATGGTGGCGACAGGA
L-P2（RHDV1)-F	GGAGGAGGAGGAAGCGGAGGAGGAGGATCTGGAAGCATGTGGAGCAGCCCC
L-P2（RHDV2)-R	CTCCGCTTCCTCCTCCTCCTGATCCTGTGCCGTTTGTGCTGC
VP60-P2(RHDV1)-F	CATGGGCGGCCGCGATATCATGTGGAGCAGCCCCC
VP60-P2(RHDV1)-R	CTGCAGGGAATTCGGATCCGGTTCCGTTGGCGGA
VP60-P2(RHDV2)-F	CATGGGCGGCCGCGATATCTGGAGCAGCCCCCGC
VP60-P2(RHDV2)-R	CTGCAGGGAATTCGGATCCTGTGCCGTTTGTGCTGC
BD-F	GGATCCGAATTCCCTGCAG
BD-R	GATATCGCGGCCGCCCATGGA

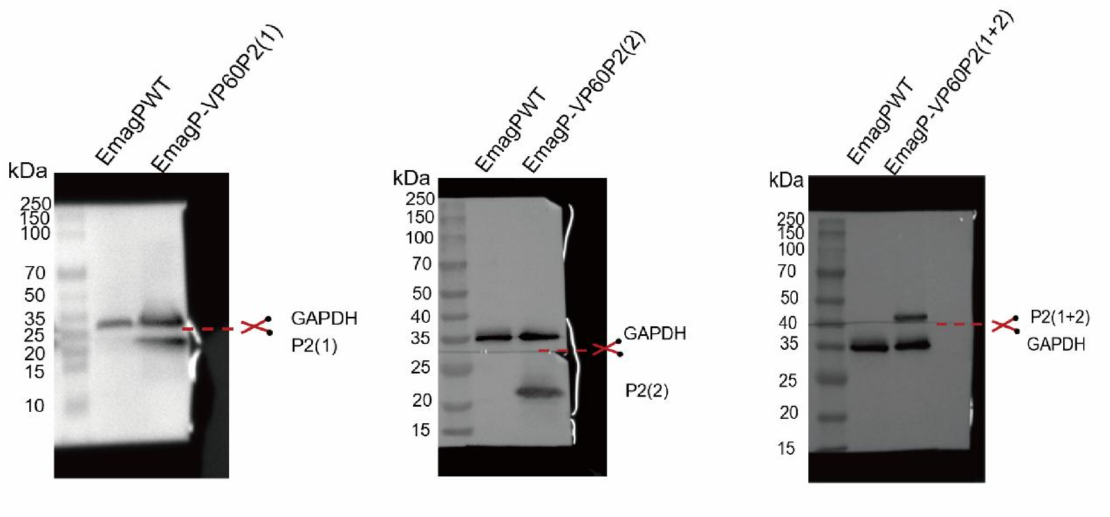

Supplementary Fig.1 Original Western blot images of P2 protein expression in recombinant EmagPWT strains.

Western blot membrane was cut according to the molecular weights of GAPDH and target protein, then separately incubated with primary antibodies: mouse anti-GAPDH antibody and rabbit anti-His tag antibody, followed by secondary antibodies: HRP-conjugated goat anti-rabbit IgG (H+L) or HRP-conjugated goat anti-mouse IgG (H+L). Western Blot analysis using rabbit anti-His-tag antibody showed specific products at ~22 kDa in EmagP-VP60P2(1) and EmagP-VP60P2(2), and ~45 kDa in EmagP-VP60P2(1+2) . Protein from EmagPWT served as a negative control.

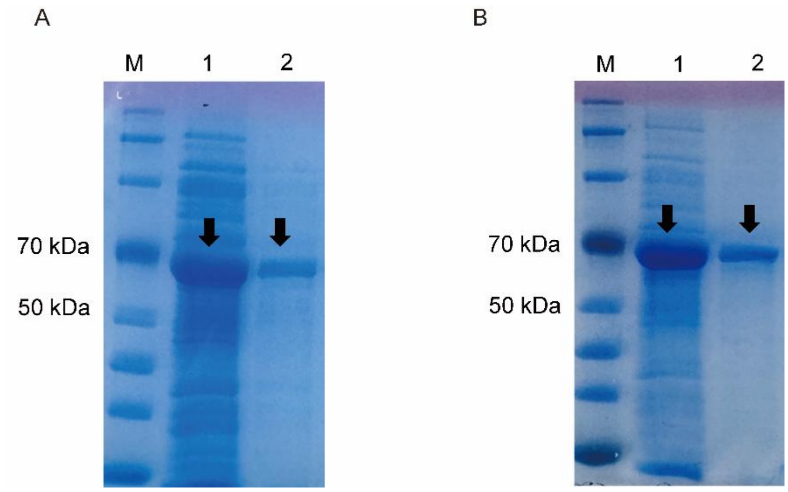

Supplementary Fig.2 Expression and purification of rRHDV1-P2 (A) and rRHDV2-P2 (B).

M: Biotides protein molecular weight standard; lane 1: Crude cell lysate of *E. coli* BL21(DE3) expressing rRHDV1-P2 (A) and rRHDV2-P2 (B) using the pMAL-C5x vector; lane 2: Purified rRHDV1-P2 (A) and rRHDV2-P2 (B). The black arrow indicates the recombinant protein.

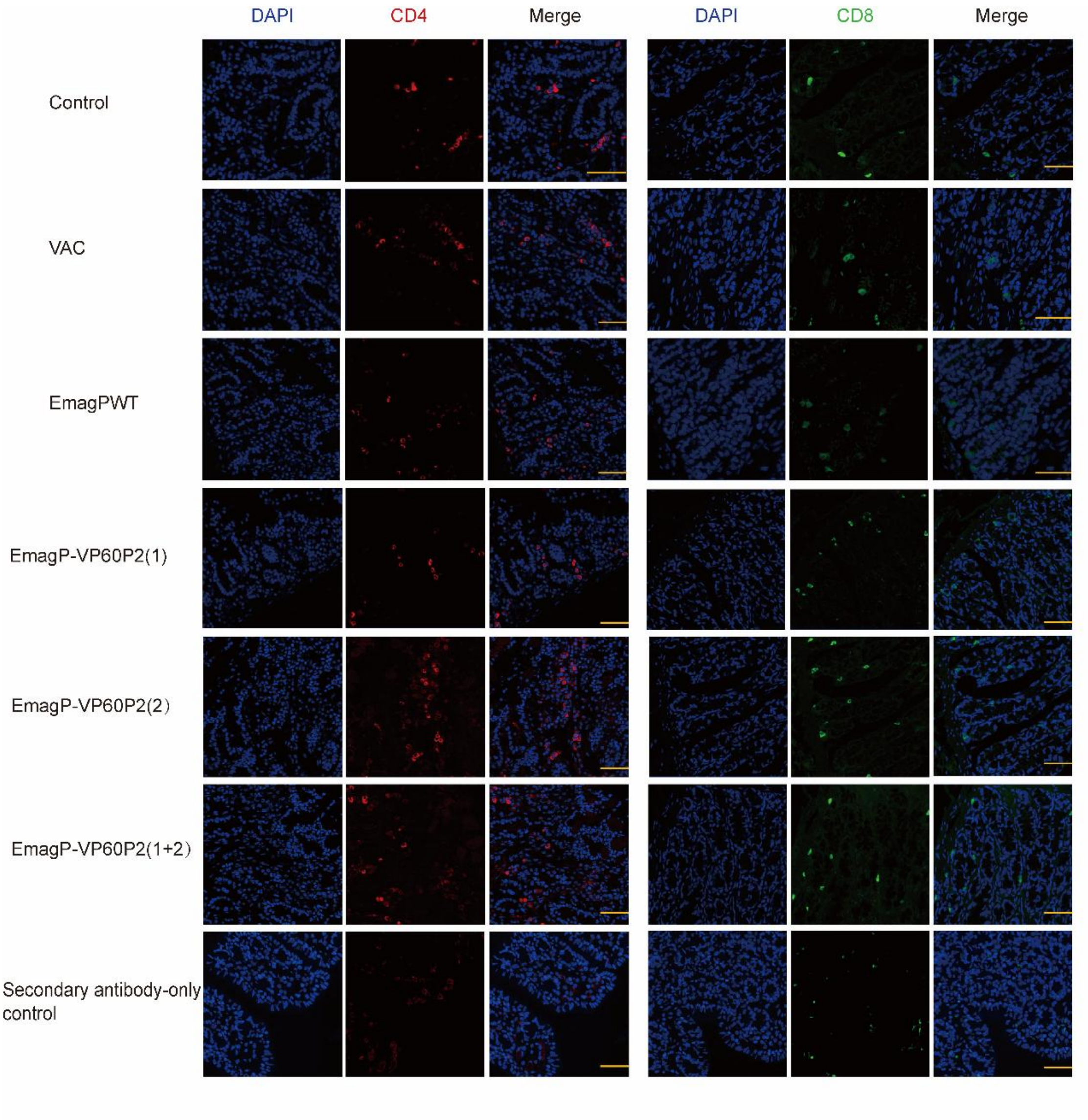

Supplementary Fig.3 Detection of CD4+ and CD8+ T cells in the ileum by immunofluorescence.

Representative immunofluorescence images show CD4+ (red) and CD8+ (green) T cell distribution in ileal tissues at 14 days post-secondary immunization. Nuclei were counterstained with DAPI (blue) (n=3). Scale bar= 50 μm.
